# Retrospective study on the toxicity induced by stereotactic body radiotherapy: overview of the reunion experience on prostate cancer in elderly patients

**DOI:** 10.3389/fonc.2024.1302001

**Published:** 2024-02-01

**Authors:** Youssef Slama, Gilles Baumont, Angelique Arcambal, Mickael Begue, Olivier Maillot, Rima Sayah, Romain Castanet, Raoul Caboche, Pedro Liberati, Hakim Slaoui, Medi Bouaziz, Olivier Borson, Nam P. Nguyen, Fabien Dutheil

**Affiliations:** ^1^ Clinique Sainte-Clotilde, Department of Radiotherapy, Groupe Clinifutur, Saint-Denis, La Réunion, France; ^2^ Clinique Sainte-Clotilde, Department of Urology, Groupe Clinifutur, Saint-Denis, La Réunion, France; ^3^ Cabinet de Radiologie Les Alizés, Saint-Denis, La Réunion, France; ^4^ Department of Radiation Oncology, Howard University, College of Medicine, Washington, DC, United States

**Keywords:** prostate cancer, elderly patients, stereotactic body radiotherapy, gastrointestinal toxicity, genitourinary toxicity

## Abstract

**Introduction:**

Prostate cancer is the fourth most commonly diagnosed cancer among men worldwide. Various tools are used to manage disease such as conventional radiotherapy. However, it has been demonstrated that large prostate volumes were often associated with higher rates of genitourinary and gastrointestinal toxicities. Currently, the improvements in radiotherapy technology have led to the development of stereotactic body radiotherapy, which delivers higher and much more accurate radiation doses. In order to *complete literature data about short-term outcome and short-term toxic effects of* stereotactic body radiotherapy*, we aimed to share our experience about gastrointestinal and genitourinary toxicities associated with* stereotactic body radiotherapy *in prostate cancer in patients over 70 years old.*

**Methods:**

We retrospectively reviewed the medical records of elderly patients with prostate cancer treated between 2021 and 2022. The elderly patients were treated with a non-coplanar robotic stereotactic body radiotherapy platform using real-time tracking of implanted fiducials. The prostate, with or without part of the seminal vesicles, was treated with a total dose of 36.25 Gy delivered in five fractions, each fraction being administered every other day.

**Results:**

We analyzed a total of 80 elderly patients, comprising 38 low-, 37 intermediate- and 5 high-risk patients. The median follow-up duration was 12 months. We did not observe biochemical/clinical recurrence, distant metastasis, or death. Grade 2 acute genitourinary toxicity was observed in 9 patients (11.25%) and Grade 2 acute gastrointestinal toxicity in 4 patients (5.0%). We did not observe any grade 3 or more acute or late toxicities.

**Conclusion:**

Over the follow-up period, we noted a low frequency of gastrointestinal and genitourinary toxicities induced by stereotactic body radiotherapy in the context of prostate cancer in elderly patients. Therefore, stereotactic body radiotherapy seems to represent a promising treatment option for elderly patients, with acceptable acute toxicity.

## Introduction

Based on prostate cancer (PCa) statistics, PCa is the 2^nd^ most commonly occurring cancer in men and the 4^th^ most common cancer overall. There were more than 1.4 million new cases of prostate cancer in 2020 (1). Nowadays, the clinical localization of the disease determines the risk level of the disease through the National Comprehensive Cancer Network (NCCN) guidelines. As per these guidelines, most patients diagnosed for PCa have low-risk or intermediate-risk disease (2). The 5-year survival rate for people diagnosed with PCa is 84% for those with local- or regional-stage PCa. However, this rate drops to 31% for those diagnosed with distant-stage *disease* (3, 4). Despite an overall 10-year survival rate of 98% across all stages, which is attributed to the high cure rate of the disease in the United States, PCa treatment is still associated with a risk of disability and co-morbidities (5). Various disease management strategies can be considered for the treatment of PCa, including definitive external beam radiotherapy (EBRT) delivered in conventional fractions of 1.8 to 2.0 Gy for 8 or 9 weeks. Given that prostate cancer exhibits a high sensitivity to higher doses per fraction due to a low α to β ratio compared to organs at risk, hypofractionated treatment with a higher dose per fraction seems to be more appropriate and more effective. Over the past 25 years, radiotherapy procedures have significantly advanced, resulting in improved precision for locating and tracking the tumor, and decreased positioning error rates in the treatments (6). This improvement has led to the emergence of Stereotactic Body Radiation Therapy (SBRT) or extreme hypofractionation. In fact, the combination of multiple fields with image guidance and SBRT allows to deliver higher and more accurate radiation doses than in the past (7–13).

Nevertheless, hypofractionation may not be beneficial for all types of tumors. In case of PCa, it could help to balance the benefits and risks by improving cure rates and reducing risks of gastrointestinal and genitourinary toxicities (14–17).

Aging affects the individual’s tolerance to ionizing radiation due to physiological changes and comorbid illnesses. Indeed, geriatric conditions can influence the normal tissue response to radiation and affect the ability of patients to complete radiation treatment and tolerate radiotherapy-related side effects (18). Of note, radiation-induced toxicities are not directly proportional to age but are more associated with the severity of the comorbidities of patients (19). Moreover, it is well established that the probability of developing PCa increases with age and that men aged 70 and older may especially experience radiation-induced toxicities (4).

Even if over the past decade, SBRT technology has been extensively used worldwide and the data collected has proved that its effectiveness and acceptability are constantly increasing, there is a lack of literature data regarding elderly patients and the short-term outcomes of SBRT, particularly potential short-term toxic effects.

The aim of this study is to share our experience regarding the short-term outcomes of SBRT in elderly patients (70 years old and older) including acute toxicity associated with the treatment in a cohort of 80 patients with various PCa risk levels (low to high-risk) treated between 2021 and 2022.

## Materials and methods

### Patient selection and characteristics

Elderly patients with PCa, for whom radiation therapy was selected as the preferred treatment in a multidisciplinary consultation meeting and who opted for SBRT over EBRT, were included in this study. The 80 patients were exclusively treated with SBRT at La Clinique Sainte-Clotilde (Reunion Island, France) for the first time and all toxicity data were collected. To enable tracking of the prostate and improve the accuracy of the dose delivery during SBRT, 3 or 4 gold seeds were inserted into the prostate transperineally or transrectally. In case of transrectal insertion, prophylactic antibiotics were administrated to the patient before and after the procedure. The fiducial markers inserted were gold anchor (0.4x10 mm) delivered through a thin needle (G22) (20, 21). Patients underwent planning computed tomography with a slice thickness of 1.5 mm at least 7 days after fiducial markers insertion. The computed tomography scan extended at least 15 cm above and below the prostate to ensure the inclusion of the testicles in the scanned volume. Additionally, a magnetic resonance imaging of the prostate was performed, specifically to delineate the urethra (22, 23). For low-risk patients, the clinical target volume (CTV) included only the prostate. However, for intermediate or high-risk patients, the CTV included the prostate and a proximal 1 cm of the seminal vesicles (24). The organs at risk (OAR) were delineated according to the recommendations of the Radiation Therapy Oncology Group (RTOG) (25). The bladder was contoured as a solid organ from base to dome. The rectum was contoured from recto-sigmoid flexure to anal verge. and the urethra from bladder to 2 cm below the prostatic apex. The bowel was countered as a “bowel bag” i.e in the space within the peritoneal cavity that could contain the bowel.

The following instructions were given to all patients to ensure an appropriate bladder and rectum preparation:

- Empty the bladder one hour before the dosimetric scanner and the radiotherapy sessions then drink 50 cl of water and avoid urinating.
- Low residue diet during the treatment phase (from the medical consultation).
- Prescription of daily laxative (from the medical consultation).
- Fasting 4 hours before the dosimetric scanner and the treatment sessions.
- Prescription of Enema 2 hours before the dosimetric scanner and the treatment sessions.

### Radiation treatment

#### Planning

The radiotherapy planning target volume (PTV) is created by adding appropriate margins to the CTV. To create the PTV, the CTV is expanded by 5 mm in all directions, except 3 mm posteriorly. This volume likely includes 1-2 mm of microscopic extracapsular spread, which helps mitigate delineation uncertainties and treatment delivery inaccuracies as reported in literature trials (26, 27). However, optimal margins for high-risk patients needed to be defined. The primary planning objective was to deliver 36.25 Gy in 5 fractions to the PTV. The plans were normalized such that 95% of the PTV volume receives at least 36.25 Gy. The dosimetric objectives to the OAR are summarized in **Table 1**.

**Table 1:** Organ at risk (OAR) dose constraints.

*Organ at risk*	*Volume*	*Dose*
***Rectum***	Maximum point dose (0.03 cc)	≤38.06Gy (105% of the prescription dose)
	Less than 3 cc	<34.4Gy (95% of the prescription dose)
	10% rectum	≤32.625Gy (90% of the prescription dose)
	20% rectum	≤29Gy (80% of the prescription dose)
	50% rectum	≤18.125Gy (50% of the prescription dose)
***Bladder***	Maximum point dose (0.03 cc)	≤38.06Gy (105% of the prescription dose)
	10% bladder	≤32.625Gy (90% of the prescription dose)
	50% bladder	≤18.125Gy (50% of the prescription dose)
***Penil bulb***	Maximum point dose	<100% of the prescription dose
	Less than 3 cc	20Gy (54% of the prescription dose)
***Femoral heads***	Less than 10 cc accrued (right-left)	20Gy (54% of the prescription dose)
	Maximum point dose	30Gy (81% of the prescription dose)
***Bowel (GETUG 14)***	D5 cc	<18.1Gy
	D1 cc	<30Gy
***Urethra***	Maximum dose	≤38.78Gy (107% of the prescription dose)

**cc** = cubic centimetre.

#### Treatment

All patient were treated with a non-isocentric robotic radiation therapy platform (CyberKnife; Accuray, Sunnyvale, CA) capable of producing rapid fall-off dose gradients with submillimeter accuracy in dose delivery (28, 29). Three or four prostate fiducials were tracked in real time, with automatic correction for translational and rotational target motion. Treatment was completed over a period of 10 to 14 days. Retrospective assessment of genitourinary and gastrointestinal functions was performed using the CTCAE V.5 scale systems at regular intervals during the first 12 months following the beginning of the treatment (end of treatment, 1, 3, 6 month and 1 year).

### Prostate-specific antigen level quantification

The blood prostate specific antigen (PSA) levels were measured before SBRT treatment and after the completion of SBRT. The PSA bounce was defined as a PSA circulating level increase of 0.2 ng/mL from the previous level measured, followed by an important decrease.

### Statistical analysis

Results were expressed as median ± standard deviation (SD), mean ± SD or median ± interquartile range when appropriate. One-way analysis of variance (ANOVA) followed by Sidak tests was assessed. Multiple comparison between groups was performed using Graph-Pad Prism 8 program (GraphPad Software, Inc.). A *p* values ≤ 0.05 was considered statistically significant.

## Results

### Distribution of patients according to tumor characteristics

Eighty elderly patients were treated between September 2021 and December 2022 with SBRT. All characteristics of patients and tumors are listed in **Table 2**. The majority of elderly patients had a prostate cancer classified as T2a and T2b stages and 72.5% of them had a Gleason score established at 3 + 3 and 3 + 4. Furthermore, this study included a majority of low and intermediate-risk patients with 5 patients considered as being at high-risk disease and 15% of patients with a PSA>20 ng/mL. It is worth noting that 58.8% of patients underwent a hormone therapy.

**Table 2:** Distribution of patients according to their PCa characteristics and their treatments.

*Parameters*	*Score/Value*	*Number of patients (%)*
***Stage***	T1a	0 (0)
	T1b	2 (2.50)
	T1c	3 (3.75)
	T2a	35 (43.75)
	T2b	33 (41.25)
	T2c	6 (7.50)
	ND	1 (1.25)
***Gleason score***	3 + 3	30 (37.5)
	3 + 4	28 (35.0)
	4 + 3	15 (18.75)
	3 + 5	1 (1.25)
	4 + 4	1 (1.25)
	4 + 5	2 (2.50)
	5 + 5	1 (1.25)
	ND	2 (2.50)
***Initial PSA levels*** ***(ng/mL)***	<10 ng/mL	44 (55.0)
	10 – 20 ng/mL	24 (30.0)
	>20 ng/mL	12 (15.0)
***NCCN Risk grouping***	Low	38 (47.5)
	Intermediate	37 (46.3)
	High	5 (6.2)
***Hormone therapy***	Yes	47 (58.75)
	Short	1 (1.25)
	No	32 (40.0)
***Age (Mean ± SD)***	76.21 ± 5.18	
***Number of patients (N)***	80	

NCCN, National Comprehensive cancer Network; ND, not disclosed; PSA, Prostate-specific antigen.

### Radiation dosimetric data

Dosimetric data were collected and listed in **Table 3**. Results show selected dose-volume histogram parameters for the rectum, the bladder and target volumes. This table also indicates the CTV volume.

**Table 3:** Dose-volume parameters for stereotactic body radiotherapy plans.

	*Parameters*	*Median ± SD (n=80)*
***CTV***	Prostate CTV volume	32.64 ± 13.86 cc
***PTV***	Volume of PTV receiving the prescription dose	95 ± 1.02%
	PTV volume	70.83 ± 20.99
	PTV_max_ dose	40.94 ± 1.07 Gy
	PTV_min_ dose	32.69 ± 2.28 Gy
***Bladder***	Bladder maximal dose (0.035 cc)	37.43 ± 0.46 Gy
	10% dose for the bladder	28.89 ± 5.99 Gy
	50% dose for the bladder	11.57 ± 3.51 Gy
***Rectum***	Rectum maximal dose (0.035 cc)	37.14 ± 0.56 Gy
	3 cc dose for the rectum	33.66 ± 2.12 Gy
	10% dose for the rectum	29.93 ± 2.66 Gy
	20% dose for the rectum	22.27 ± 3.07 Gy
	50% dose for the rectum	10.43 ± 3.89 Gy

cc, cubic centimeter; CTV, clinical target volume; PTV, planning target volume.

**Figure 1:**
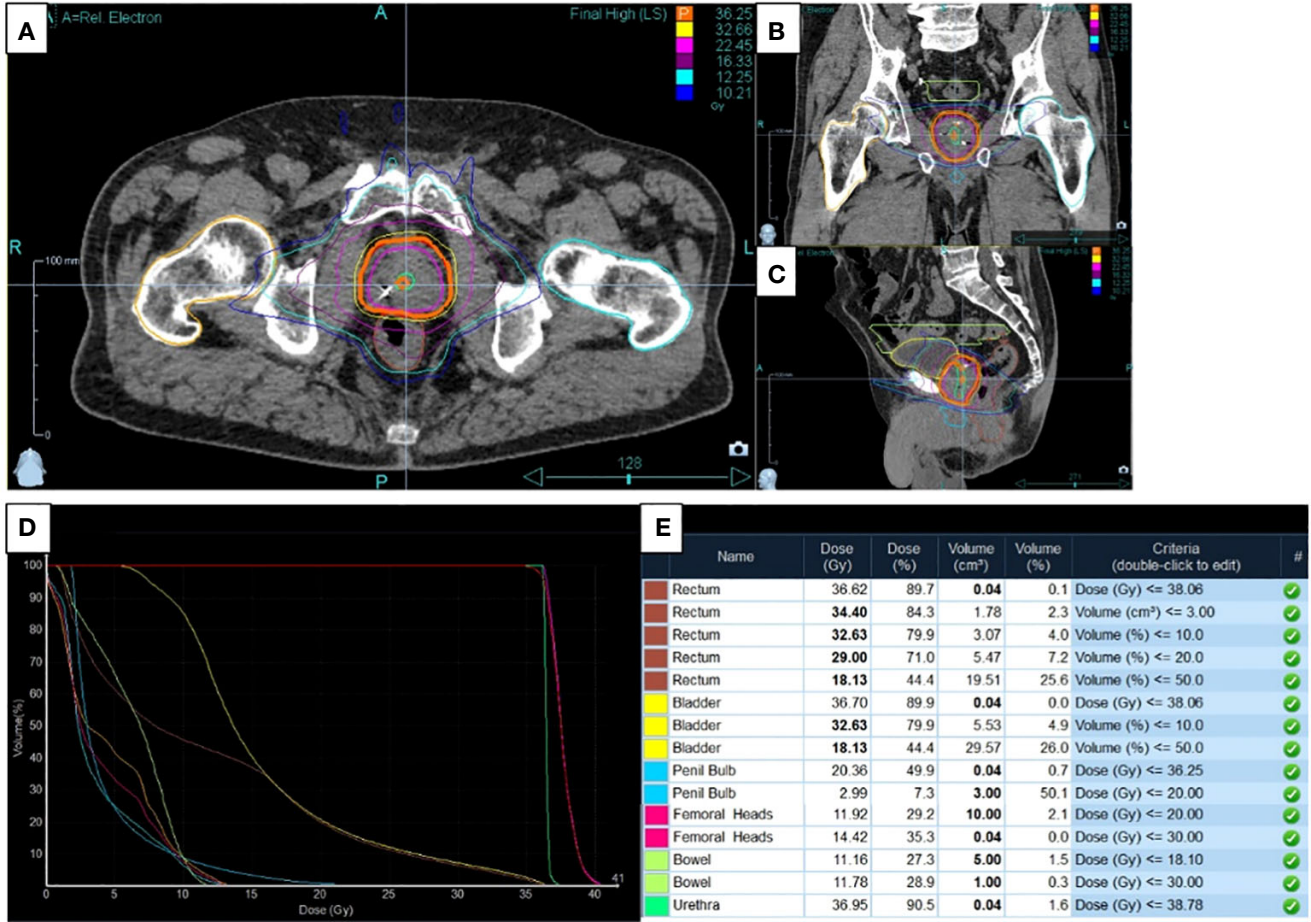
Typical dose distribution, dose-volume and dosimetric data for application of radiotherapy treatment on prostate cancer. **(A)** The axial, **(B)** sagittal and **(C)** coronal views were presented concerning dose distribution as well as **(D)** the dose-Volume Histogram and **(E)** the corresponding dosimetric validation table.

The typical dose distribution for radiotherapy treatment of prostate patients are represented in **Figure 1** with the axial (**Figure 1A**), sagittal (**Figure 1B**) and coronal (**Figure 1C**) views. The typical Dose-Volume Histogram is represented on **Figure 1D** as well as the corresponding dosimetric validation table (**Figure 1E**).

### Genitourinary and gastrointestinal toxicities reported over time

**Figure 2:**
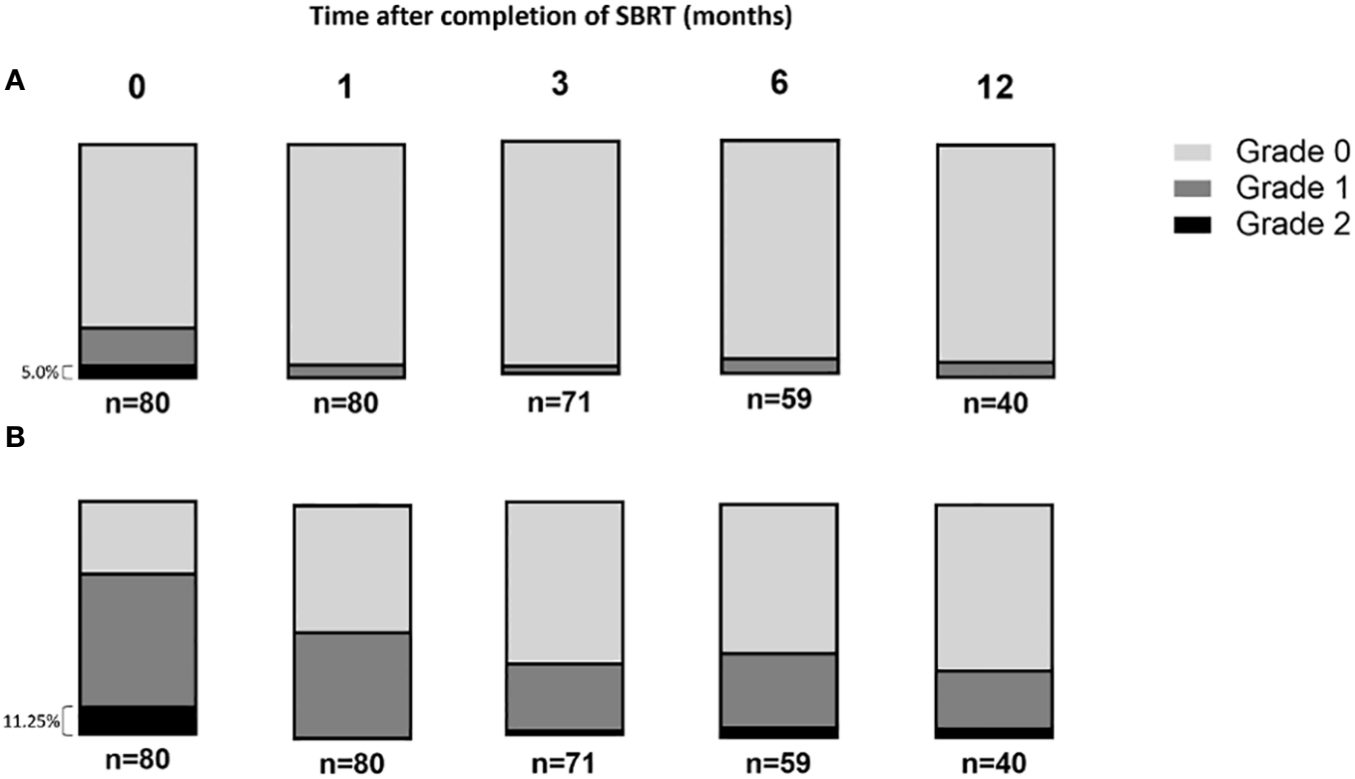
The rate of acute gastrointestinal and genitourinary toxicities after prostate stereotactic body radiotherapy scored following Radiation Therapy Oncology Group score. **(A)** The gastrointestinal and **(B)** genitourinary toxicities were presented overtime after the completion of SBRT protocol.

Toxicity induced by SBRT was assessed by gathering patients’ feedback (**Figure 2**). The reported toxicity, as measured on the CTCAE V.5 scale, was low; the gastrointestinal and the genitourinary grade 2 toxicity occurrence after treatment was 5% (**Figure 2A**) and 11.25%, respectively (**Figure 2B**). Data indicated that genitourinary toxicity became more significant over time than gastrointestinal toxicity. Moreover, two patients reported a grade 3 genitourinary toxicity at the vesical globe level.

### Correlation between reported toxicities and dose-volume parameters

**Figure 3:**
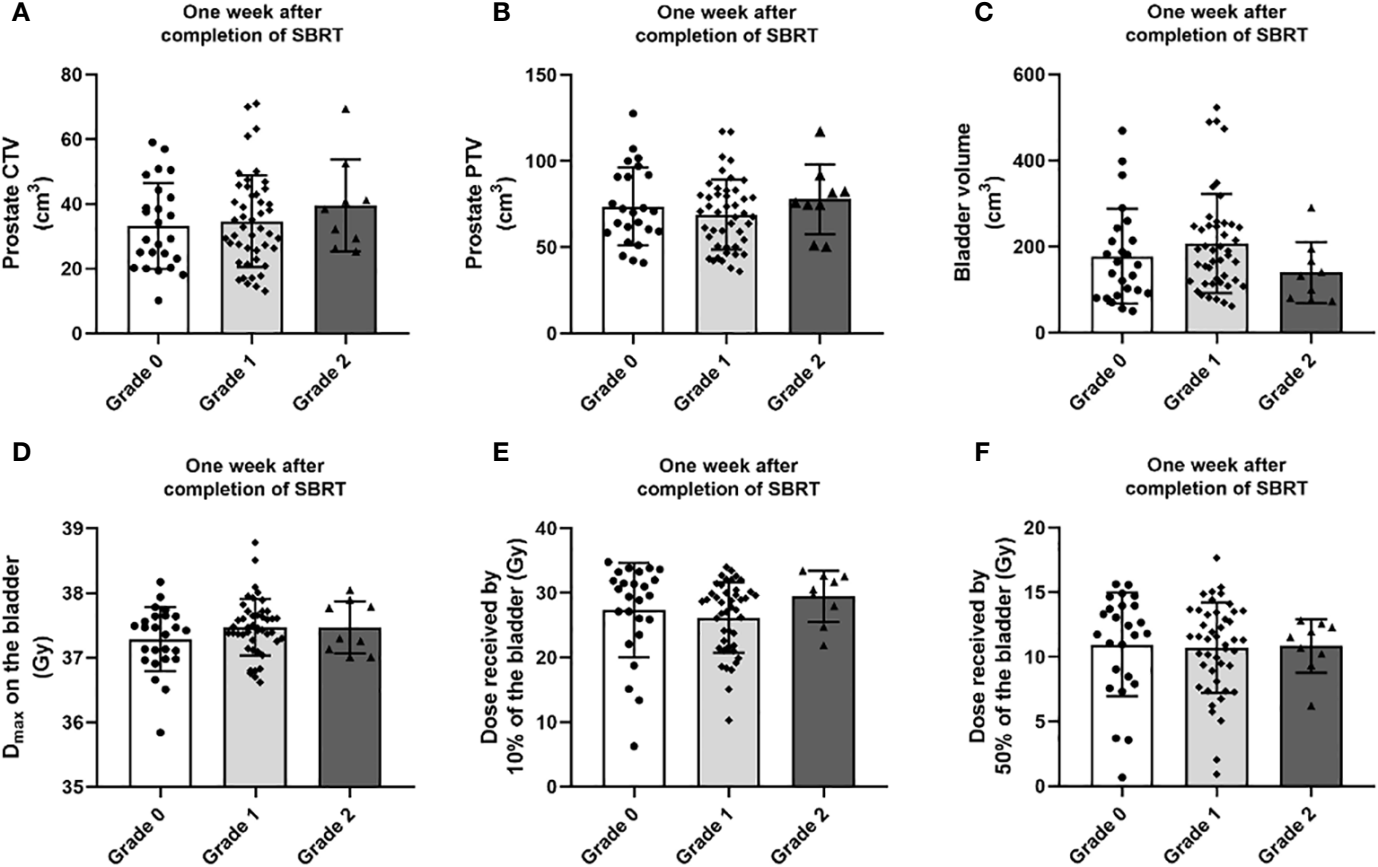
Distribution of **(A)** CTV, **(B)** PTV, **(C)** bladder volume, **(D)** maximum dose (dose in a volume of 0.035 cc) to the bladder, **(E)** the dose received by 10% of the bladder volume and **(F)** the dose received by 50% of the bladder volume by genitourinary toxicity grade occurring one week after the end of the SBRT treatment. Data are expressed as mean ± SD.

The genitourinary toxicity grades were determined by gathering patients’ feedback over time following SBRT treatment. As shown in **Figure 2**, most of the toxicities reported by the patients were genitourinary. Therefore, we analyzed the dose-volume data to investigate whether these toxicities could be predicted and correlated with CTV, PTV, and bladder volume, as well as the doses received by the bladder (**Figure 3**). No difference was observed between groups of grades 0, 1 or 2 regarding the prostate CTV, PTV (**Figures 3A, B**) and bladder volume (**Figure 3C**), one week after the end of SBRT.

On this cohort, we did not observe any correlation between the toxicity recorded 1 week after the end of SBRT and the doses received by the bladder (**Figures 3D-F**).

### Prostate-specific antigen analysis

All elderly patients selected for the present study were included in the post-treatment analysis of prostate specific antigen. The data presented in **Table 2** shows that 58.8% of patients had concomitant androgen deprivation therapy. The PSA levels quantified before the start of treatment were 19.03 ± 39.69 within a range of [0.230 – 266.00] for 80 patients.

**Figure 4:**
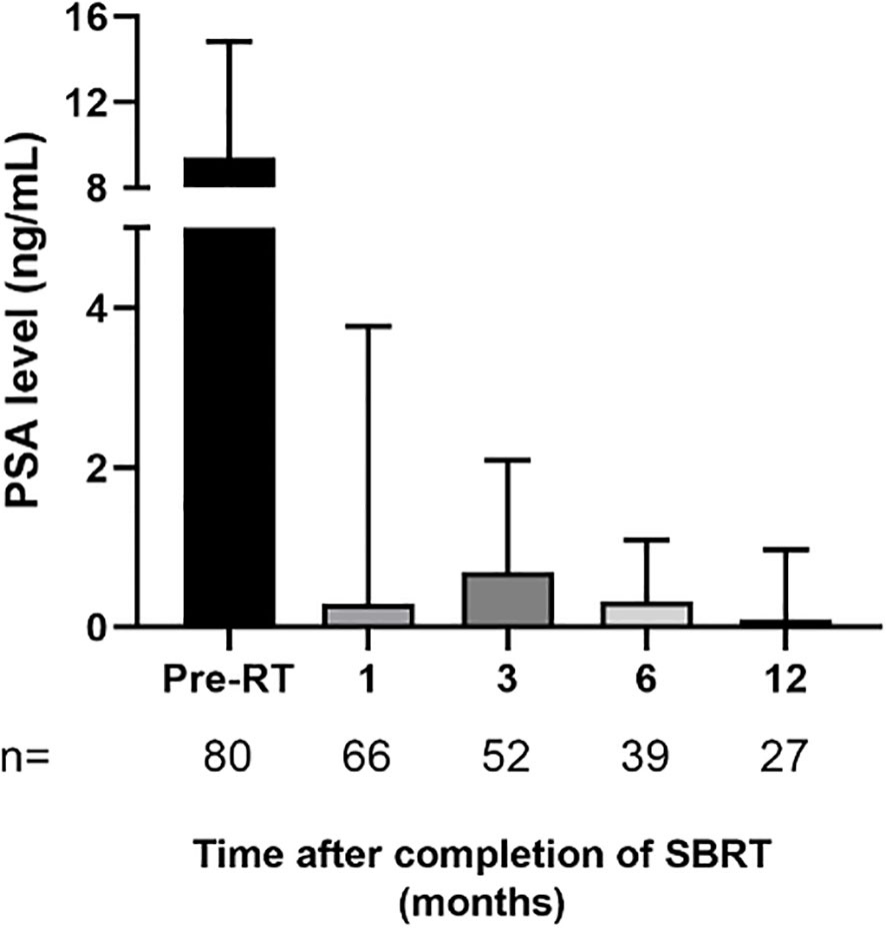
Patient level of prostate-specific antigen (PSA) after completion of prostate stereotactic body radiotherapy. Data are expressed as median ± interquartile range. The number of patients corresponding to PSA levels quantified at each period is specified on figure histogram. *Abbreviations:* ***pre-RT*** *= pre-radiotherapy.*.

The median follow-up time was 12 months and we observed a gradual decline of the median PSA level over time (**Figure 4**). Indeed, the 6 months post-treatment median PSA has dropped to 0.33 ng/ml. At 6 months after treatment, 20% of patients exhibited a temporary rise in PSA levels, followed by a subsequent decrease to the previous levels. However, PSA outcomes with such a short follow-up period should be interpreted with caution and will need to be reassessed when the median follow-up period approaches 5 years.

## Discussion

The aim of this retrospective study was to report the toxicity data collected from 80 elderly patients with PCa and treated with the SBRT technique using the Cyberknife system. No instances of biochemical or clinical recurrence, distant metastasis occurrence or death were observed during the follow-up period. Acute gastrointestinal and genitourinary toxicities observed in this study were not correlated with the calculated dose levels received by the bladder or the rectum. Two patients experienced grade 3 toxicity level during the SBRT treatment, which led to the interruption of their treatment.

Importantly, geriatric conditions may affect response to radiation and studies reported that comprehensive geriatric assessment (CGA) can help to predict the occurrence of acute radiation-induced toxicities for patients treated for PCa or Head and neck cancers (30–32).

In the present study, we did not perform a CGA before SBRT, which represents a limitation of our study. Recently, studies tried to predict tolerance of radiotherapy by proceeding to CGA to identify predictors of reduced Quality of Life (QoL) and occurrence of toxicities. Nevertheless, some studies on cohorts of prostate cancer elderly patients using conventional or hypofractionated radiotherapy demonstrated the lack of sensitivity of CGA outcome and did not find predictive factors to determine toxicities or impaired QoL following radiotherapy (33–35). Indeed, screening tools need to be more experienced.

PCa incidence risk increases with age and it seems crucial to pay attention to acute and or late radiation-induced toxicities for elderly patients after the completion of their radiotherapy protocol. However, literature data about the side effects of radiotherapy are more associated with protocols using EBRT than those using SBRT and the level of evidence in older patients is limited. Thus, we chose to focus on SBRT-induced toxicities.

Currently, SBRT technology is a new technique that presents a significant benefit for PCa treatment. As demonstrated in literature, results of SBRT are encouraging, supporting its use for PCa treatment (36, 37).

In fact, several phase III randomized non-inferiority trials have mentioned that SBRT allowed tumor control without providing serious toxicities (13, 38). Our study demonstrated that SBRT was well tolerated in elderly patients; however, a longer follow-up period would be necessary to assess the real effect of the treatment. Acute grade 2 genitourinary toxicity was reported for 21 patients (13.6%). The frequency of acute genitourinary toxicity reported in previous Phase II or III studies was within a range of 20.2 to 35.3%. Thus, the frequency of these toxicities found out in our study may seem low compared to literature data (13, 38, 39). This difference could be explained by our strict adherence to the bladder dose constraints recommended by RTOG, in our treatment plans. Similarly, our study found a lower incidence of gastrointestinal toxicities compared to the levels commonly reported in the literature for prostate cancer patients undergoing SBRT. In fact, the 1-year cumulative incidence rate of grade 2 gastrointestinal toxicities reached 4% compared to 14% in other studies (40–42).

Interestingly, it has been demonstrated that moderate hypofractionated RT by helical tomotherapy used to treat patients aged ≥ 75 years with localized prostate cancer, induced acceptable acute and late toxicity. As observed in our study using extreme hypofractionated RT, Cuccia et al. did not observe a significant difference in urinary and bowel function of patients being treated by moderate hypofractionated RT (43).

Moreover, we did not observe any post-treatment grade 3 toxicity in our patients, unlike other studies which reported a toxicity of grade ≥ 3 for 1 to 2% of patients (37, 44). This low frequency of gastrointestinal toxicities could be also explained by the strict compliance with the dose constraints to the organs at risk. The low levels of toxicity in our study may be also attributed to other factors. First of all, several publications have demonstrated that image guided radiotherapy is associated with a lower level of genitourinary and gastrointestinal toxicities compared to non-image-guided radiotherapy. This may be attributed to the smaller positioning errors, thereby avoiding unnecessary irradiation of the healthy surrounding organs. Moreover, the image guidance allows for the reduction of radiotherapy planning margins, resulting in delivering lower doses to the normal tissue (45–47). In addition, the dose fall-off resulting from the use of multiple noncoplanar beams produced by the Cyberknife is sharper than in conventional techniques. Besides, the difference in the alpha/beta ratios between prostate and rectum may have helped to improve the therapeutic balance in our favor. In fact, we may be able to achieve the same cure rates with lower toxicity to the rectum which has a lower fractionation sensitivity compared to prostate cancer cells.

Biochemical response rates for prostate SBRT have been published in several trials, the largest being a trial with a cohort of 1100 patients treated within eight independent US institutions using similar protocols with doses ranging from 35 to 40 Gy delivered in 5 fractions (40).

The biochemical response rate at 5-year follow-up was 95.2% for low risk and 84.1% for intermediate risk patients (including Gleason 4 + 3) and 86% of patients did not receive androgen deprivation therapy. The authors noted that out of a total cohort of 49 patients who met the criteria for biochemical failure, 9 patients experienced a mild PSA rebound but remained biochemically controlled. A PSA rebound is a recognized but poorly understood phenomenon occurring after prostate irradiation, and is observed in 20% of the patients in our study. It can persist for several years after SBRT treatment (48).

In this context, it is important for radiation oncologists to be aware of this phenomenon to avoid subjecting patients to unnecessary examinations.

Our study was also limited by its retrospective design and low sampling. Few patients had associated comorbidities, such as diabetes or a history of cardiovascular disease requiring supplemental medication. So, we could not include the confounding factors in our study. It should be noted that this study only presents preliminary results. In particular, an important aspect to consider would be the evaluation of late toxicities, such as hematuria and rectal hemorrhage which are commonly observed within 2 years following SBRT treatment. Therefore, the short-term findings reported in our study should be interpreted with caution. A longer follow-up is necessary, especially to assess treatment effectiveness and late toxicities.

## Conclusion

The retrospective results obtained from this cohort showed that SBRT treatment for PCa in elderly patients is well tolerated and provides an early biochemical response and good efficacy over a period of one year for patients from Reunion Island. This is in line with the data from randomized trials such as PACE B and HYPO-RT trials, which showed the benefit of SBRT for men with low- and intermediate-risk prostate cancer. Although the treatment is generally well-tolerated by the patients, the occurrence of gastrointestinal and genitourinary toxicities remains a significant problem.

## Data availability statement

The raw data supporting the conclusions of this article will be made available by the authors, without undue reservation.

## Ethics statement

This retrospective clinical study was evaluated and approved bythe Ethics Committee of Research at the University of Montpellier on 09 May 2023 (UM 2023-013). All data collected were obtained during routine clinical practice and all authors conducted this study by respecting the ethical standards of their respective institutions as laid down in the 1964 Declaration of Helsinki. All patients were informed about the use of their individual personal data and gave their consent to participate in the present study.

## Author contributions

YS: Writing – original draft, Data curation, Methodology, Supervision. GB: Validation, Writing – review & editing. AA: Methodology, Writing – original draft, Formal analysis, Resources. MiB: Methodology, Writing – original draft. OM: Validation, Writing – review & editing. RS: Methodology, Writing – review & editing. RoC: Conceptualization, Writing – original draft. RaC: Data curation, Writing – review & editing. PL: Writing – original draft, Data curation. HS: Methodology, Writing – review & editing. MeB: Data curation, Writing – review & editing. OB: Data curation, Writing – original draft. NN: Writing – original draft. FD: Writing – original draft, Data curation, Methodology, Supervision.
